# *Equisetum arvense* L. Extract Induces Antibacterial Activity and Modulates Oxidative Stress, Inflammation, and Apoptosis in Endothelial Vascular Cells Exposed to Hyperosmotic Stress

**DOI:** 10.1155/2018/3060525

**Published:** 2018-02-14

**Authors:** Annamaria Pallag, Gabriela Adriana Filip, Diana Olteanu, Simona Clichici, Ioana Baldea, Tunde Jurca, Otilia Micle, Laura Vicaş, Eleonora Marian, Olga Soriţău, Mihai Cenariu, Mariana Mureşan

**Affiliations:** ^1^Department of Pharmacy, Faculty of Medicine and Pharmacy, University of Oradea, 29 Nicolae Jiga Street, 410028 Oradea, Romania; ^2^Department of Physiology, “Iuliu Haţieganu” University of Medicine and Pharmacy, 1-3 Clinicilor Street, 400006 Cluj-Napoca, Romania; ^3^Department of Preclinical Disciplines, Faculty of Medicine and Pharmacy, University of Oradea, 10 Piata 1 Decembrie Street, 410073 Oradea, Romania; ^4^Departments of Radiobiology and Tumor Biology, Oncology Institute “Prof. I. Chiricuta”, 34-36 Republicii Street, 400015 Cluj-Napoca, Romania; ^5^Department of Biochemistry, University of Agricultural Sciences and Veterinary Medicine, Calea Manastur 3-5, 400372 Cluj-Napoca, Romania

## Abstract

**Background:**

The antimicrobial activity of the *Equisetum arvense* L. extract and the mechanisms involved in the *in vitro* effects on endothelial vascular cells exposed to hyperosmotic stress were evaluated.

**Methods:**

Antimicrobial activity was evaluated by disk diffusion method and minimum inhibitory concentration (MIC) determination, and oxidative stress, inflammation, and apoptosis, in pretreatment with *Equisetum arvense* L., caffeic acid, and cathechin, were quantified.

**Results:**

The results have shown that *Equisetum arvense* L. exhibited antibacterial effects only on pathogenic gram-positive cocci. The modulatory activity of *Equisetum arvense* L. on endothelial cells exposed to hypertonic medium was different and depended on the concentration used. Low concentrations of tested compounds exerted antioxidant effect and diminished the activity of caspase-8 and also increased I*κ*B expression while in high doses, *Equisetum arvense* L. was prooxidant, induced apoptosis, and decreased IL-6 secretion.

**Conclusions:**

These experimental findings suggest that *Equisetum arvense* L. has antibacterial effects on gram-positive cocci and, administered in low dose, may be a new therapeutic approach for diseases associated with hypertonic conditions or oxidative stress and apoptosis.

## 1. Introduction

Worldwide, research in the past decades has shown an increased interest in the phytochemical products and plant extracts, due to frequent use in the prevention and treatment of some diseases. Several studies have demonstrated that the antioxidants found in plants are of major interest to medicine, owing to the fact that they protect the organism against oxidative stress, generated in the context of some diseases: atherosclerosis, ischemic cardiac disease, cancer, Alzheimer disease, Parkinson disease, aging, and even in infectious diseases [1]. Experimental data have shown that polyphenols can offer an indirect protection by activating the antioxidant transcription through antioxidant-responsive elements (AREs) from the promoter regions of the genes induced by oxidative stress [2]. In addition, polyphenols can modulate the cell signaling involved in cell proliferation [3] and cell cycle progression [4] while also inhibiting inflammation and promoting the apoptosis of damaged cells [5].

*Equisetum arvense L* (field horsetail) is a perennial fern from the *Equisetaceae* family. It has a yellowish nonphotosynthetic spore-bearing fertile stem, produced in early spring. The green, photosynthetic, heavily branched sterile stems are produced in late spring and persist to late autumn. The sterile stem is the medicinal product of the plant (*Equiseti herba)* mentioned in European Pharmacopeia (Ph. Eur. 8) [6]. The most widely known phytochemical compounds of *Equisetum arvense* L. are flavonoids, phenolic acids, alkaloids, phytosterols, tannins, and triterpenoids [7].

Several studies have described different biological effects of *Equisetum arvense* L. extract or tea with natural extract, such as antioxidant, anti-inflammatory, antibacterial, antifungal, vasorelaxant, neuro and cardio protectors [8, 9], and antiproliferative properties [7, 10].

Cardiovascular diseases are a leading health problem worldwide [11]. 31% of all deaths are caused by cardiovascular conditions and over 75% of deaths in low-income and middle-income countries can be accredited to heart disease [12]. High salt intake, obesity, aging, impaired glucose tolerance, and diabetes are just some of the factors that correlate positive with inflammation, coagulations alterations, and elevated of extracellular fluid osmolarity found in cardiovascular diseases. In the center of these processes, the microlesion of vascular endothelium under the influence of smoking, hypertension, hyperlipidemia, hyperglycemia, hyperlipoproteinemia, hyperhomocysteinemia, and so forth [13, 14] is the key factor. The vascular endothelial dysfunction is characterized by loss of physiological function such as vasodilation, fibrinolysis, and antiplatelet activity [15]. An imbalance of the factors responsible for modulating vascular tone appears especially with reduced synthesis and release of nitric oxide (NO) with impaired vasodilation and an increase in angiotensin II and endothelin levels, with the onset of abnormal vasoconstriction.

An increased extracellular osmolarity has many negative effects on the endothelium: promotes water flux out of the cells, triggers cell shrinkage and intracellular dehydration [16], increases oxidative stress, and affects protein structure and function in particular enzyme activity with consequences on the structure of the cytoskeleton and nucleus [17]. DNA lesions trigger activation of growth arrest and DNA damage-inducible genes including p53 [17] and promote the apoptosis, according to the degree of osmotic imbalance.

In order to equalize the intracellular and extracellular pressure, the cells develop an adaptive response to osmotic stress by induction of genes responsible for synthesis and transports of “compatible osmolytes.” Many of them, such as heat shock protein, antioxidant enzymes, and adhesion molecules, are required for preserving the protein structure and function [18] and for increasing the protein stability [17]. Moreover, as a response to hyperosmotic stress, the cells express the nuclear factor of the activated T cells-5 (NFAT5), a member of the nuclear transcription factor- (NF-) *κ*B/Rel family of transcription factors, and stimulate the releasing of proinflammatory cytokines [19] such as interleukin-6 (IL-6) and tumor necrosis factor alpha (TNF-*α*) by the activated macrophages, T-lymphocytes, smooth muscle cells, and endothelial cells. Reactive oxygen species (ROS) are highly reactive molecules which cause, on one hand, lipid peroxidation, DNA damage, and protein oxidation [20] and, on the other hand, activate stress-activated protein kinases (ERK), protein kinase B, or Akt kinases and NF-*κ*B.

The study of the cytotoxicity and antimicrobial activity of plant extracts is important for their clinical application in medical practice. Bacterial infections, especially those that form biofilm or are associated with antibiotic failure, require the finding of agents with complementary action or synergic effect with antibiotherapy. *Staphylococcus aureus* is known as an etiologic agent for many skin and mucous infections, especially those caused by methicillin-resistant *S. aureus* (MRSA) at immunocompromised patients or postoperative [21]. *E. coli* is frequently described as a cause of chronic and recurrent urinary infection [22] while *C. albicans* is the most pathogenic species in humans, isolated from biofilms formed on implanted medical devices [23] or is involved in oral candidiasis, both in adult and in children [24]. Several studies have shown that polyphenols from plants can be an alternative to antibiotics against microbial pathogens [25–27].

Starting from the previously obtained results, we aimed to test *Equisetum arvense* L. extract for its antimicrobial activity and for anti-inflammatory, antioxidant, and antiapoptotic properties on endothelial cell cultures. The plant used comes from nonpolluted areas of Bihor County, the harvest time being the months of April and May, when we obtained the highest values of phenolic compound concentration. The effect against gram positive, gram negative, and *Candida albicans* by disc diffusion test and minimum inhibitory concentration (MIC) was evaluated. Oxidative/antioxidative status and inflammatory status in addition with caspase-3 and 8 activities and their interaction with two doses of *Equisetum arvense* L. extract, on osmotic stress *in vitro*, were investigated.

## 2. Material and Methods

### 2.1. Plant Collection and Sample Preparation

In our previous research, the plant collection and sample preparation were described [28]. Thus, the sterile stems of *Equisetum arvense* L. collected in May 2015 (Bihor County) was dried at 40°C, for 96 h, and used for preparation of lyophilized extracts. The extraction of 10 g of sterile stems was made with 100 ml 70% aqueous ethanol for 20 minutes. After centrifugation (10 minutes, 3000*g*), the extract was evaporated to dryness and then was transferred to a sample via lyophilized extract. Shimadzu SCL-POA reversed-phase high-performance liquid chromatography (HPLC) system consisted of a LC-10ADVP pump equipped with SPD-10AVP Diode Array detector was used to assay compositions of phenolic compounds. The column Kintex 5u RPC 18 and mobile phase composed of 0.05% formic acid and 0.05% formic acid-acetonitrile (50 : 50 *v*/*v*) were used. The antioxidant activity was evaluated by CUPRAC assay, DPPH method, and FRAPS method. Total polyphenol content was determined with the Folin–Ciocalteu method.

### 2.2. Chemicals

2-Thiobarbituric acid and Bradford reagent were obtained from Merck KGaA (Darmstadt, Germany), while absolute ethanol and n-butanol were purchased from Chimopar (Bucharest, Romania). ELISA tests for evaluation of caspase-3, caspase-8, IL-6, and TNF-*α* were obtained from R&D Systems (Minneapolis, MN, USA). NF-*κ*B, phospho-NF-*κ*B p65 (Ser 536), (93H1), I*κ*B*α*, and phospho-I*κ*B*α* (Ser 32/36) were purchased from Cell Signaling Technology Inc., Danvers, USA.

### 2.3. The Antimicrobial Activity

Antimicrobial activity of *Equisetum arvense* L. was evaluated by disk diffusion method using the standard methodology [29] and by determination of minimum inhibitory concentrations (MICs).

#### 2.3.1. Microorganisms

In our study, we used the following test organisms: reference microbial strains—*Staphylococcus aureus* (ATCC 29213), *Streptococcus pneumoniae* (ATCC 49619), *Escherichia coli* (ATCC 25922), *Pseudomonas aeruginosa* (ATCC 27853), and *Candida albicans* (ATCC 90029) (Microbiologics, USA) and human clinical isolates—*Staphylococcus aureus*, *Staphylococcus epidermidis*, *Streptococcus pyogenes*, *Streptococcus agalactiae*, and *group G beta-hemolytic streptococcus*.

#### 2.3.2. Disc Diffusion Test

For staphylococci, *Escherichia coli*, and *Pseudomonas aeruginosa* isolates, Mueller-Hinton Agar (Oxoid) was used and for streptococcal strains, Mueller-Hinton 2 agar + 5% sheep blood (BioMerieux). *Candida albicans* was plated onto Sabouraud Gentamicin Chloramphenicol 2 agar (BioMerieux).

Inoculums were prepared by direct colony suspension in salina from 20–24 h growth from Columbia agar + 5% sheep blood (Biomeriex) for bacterial strains and from Sabouraud Gentamicin Chloramphenicol 2 agar (BioMerieux) for *Candida albicans*, equivalent to a 0.5 McFarland standard. Each strain was plated onto the appropriate culture media with a sterile cotton swab, and plates were dried for 10–15 minutes. Sterile filter paper discs of 6 mm diameter (HiMedia Laboratories) saturated with 20 *μ*l of *Equisetum arvense* L. extract were placed onto inoculated plates. After overnight incubation, at 37°C, inhibition zone diameters were measured in millimeters. Filter papers impregnated with distilled water (20 *μ*l) were used as negative controls, and penicillin (10 U, Oxoid), vancomycin discs (30 *μ*g; Oxoid), ofloxacin discs (5 *μ*g; Oxoid), meropenem discs (10 *μ*g; Oxoid), and fluconazole (25 *μ*g; BD BBL) were used as positive controls. Each test was performed in triplicate and mean values were selected.

#### 2.3.3. Minimum Inhibitory Concentration (MIC) Determinations

The MIC is defined as the lowest concentration of a drug that will inhibit the visible growth of an organism after overnight incubation. We used Broth dilution MIC's—macrodilution for the following test organisms: reference microbial strain—*Staphylococcus aureus* (ATCC 29213), *Streptococcus pneumoniae* (ATCC 49619), *Streptococcus pyogenes group A* (ATCC®12384™ Culti-Loops™) and human clinical isolates—*group G beta-hemolytic streptococcus* and *Staphylococcus aureus.* For staphylococci isolates, Mueller-Hinton Broth (Oxoid) was used and for streptococcal strains, Mueler-Hinton Broth plus 5% sheep blood (BioMerieux). We used 75 × 12 mm sterile capped tubes, in two rows for each microbial strain to cover the range of *Equisetum arvense* L. The first tube, an inoculated tube, controlled the adequacy of the broth to support the growth of the organism, while the second tube was used to check the sterility (an uninoculated tube).

From 200 mg/ml *Equisetum arvense* L. extract, five dilutions were prepared as follows: 100 mg/ml, 50 mg/ml, 25 mg/ml, 12.5 mg/ml, and 6.25 mg/ml. Inoculums were prepared by direct colony suspension in salina from 20–24 hours grown from Columbia gar supplemented with 5% sheep blood (BioMerieux), equivalent to a 0.5 McFarland standard. 0.1 ml inoculum for each tube was used and all the tubes were incubated at 37°C overnight.

### 2.4. *In Vitro* Experimental Data

#### 2.4.1. Cell Culture

Commercial human umbilical vein endothelial cells (HUVEC) were bought from the European Collection of Cell Cultures (ECACC, Porton Down, Salisbury, UK) and multiplied in RPMI medium, supplemented with 10% fetal calf serum (FCS), gentamicin 50 *μ*g/ml, and amphotericin 100 *μ*g/ml (Biochrom AG, Berlin, Germany) in a humidified CO_2_ incubator at 37°C. Cell cultures in the 23rd to 26th passages were used. The analysis of surface markers was performed by flow cytometry (BD FACS Canto II flow cytometer, Becton Dickinson & Company, Franklin Lakes, NJ, USA) and used different monoclonal antibodies (ICAM-1, CD29, CD34, CD73, CD90, and CD105).

Cells seeded at a density of 10^4^/cm^2^ in cell culture Petri dishes (TPP) were settled for 24 h in medium then exposed for 24 h to either *Equisetum arvense* L. extract (25 or 2.5 *μ*g/ml), caffeic acid (10 or 1 *μ*g/ml), and cathechin (10 or 1 *μ*g/ml), as positive controls; afterwards, the cells were washed 3 times with PBS and incubated for 24 h either in normotonic (137 mmol/l) or hypertonic conditions (200 mmol/l). Cells were collected by scraping and treated with a lysis buffer containing IGEPAL-Nonidet 1% (Sigma) and 1% protease inhibitor complex (Sigma) in PBS for 1 h, on ice. The protein content was determined by the Bradford method (Bio-Rad, USA). All the experiments were performed in triplicate.

#### 2.4.2. Cell Viability Testing

To explore the cytotoxicity of *Equisetum arvense* L. extract, the 3-(4,5-dimethylthiazol-2-yl)-5-(3-carboxymethoxyphenyl)-2-(4-sulfophenyl)-2H-tetrazolium (MTS) method was employed. Human umbilical vein endothelial cells (HUVECs), seeded at a density of 10^4^/well in ELISA 96-well microtitration flat bottom plaques (TPP, Switzerland), were allowed to settle for 24 h in medium, then treated with different concentrations of extract in medium ranging from 6.5 to 5000 *μ*g/ml, for 24 h. Afterwards, the viability assays were performed. The viability testes were done using CellTiter 96® AQueous Non-Radioactive Cell Proliferation Assay as indicated by the producer. The optical density values were read at absorbance of 490 nm using an ELISA plate reader (Tecan, Männedorf, Switzerland) for MTS. All the experiments were conducted in triplicate. Untreated cultures exposed to medium were used as controls.

#### 2.4.3. Oxidative Stress Assessment

Lipid peroxides were determined using the fluorimetric method with 2-thiobarbituric acid (TBA) described by Conti et al. [30] and were expressed as malondialdehyde (MDA) equivalents.

#### 2.4.4. Inflammation and Apoptosis

Inflammation was quantified by measurement of IL-6 and TNF-*α* levels and apoptosis by evaluation of caspase-3 and caspase-8 activities. In addition, the expressions of total NF-*κ*B protein and the active form (phospho-NF-*κ*B), I*κ*B, and phospho-I*κ*B were evaluated by Western blot in cells exposed to low doses of the three compounds used for treatment. IL-6, TNF-*α*, the human active caspase-3 immunoassay kits, and caspase-8 colorimetric assay kit were purchased from R&D Systems Inc. (USA). Cell extracts were treated as indicated by the producer, readings were done at 450 nm with correction wavelength set at 540 nm, using an ELISA plate reader (Tecan, Switzerland).

For Western blotting, 20 *μ*g protein lysates/lane were separated by electrophoresis on SDS PAGE gels and transferred to polyvinylidene difluoride membranes, using Bio-Rad Mini-PROTEAN system (Bio-Rad). Blots were blocked and then incubated with antibodies against NF-*κ*B, pNF-*κ*B, I*κ*B, and pI*κ*B, then further washed, and incubated with corresponding secondary HRP-linked antibodies (1 : 1500) (Santa Cruz Biotechnology). Proteins were detected using Supersignal West Femto Chemiluminescent substrate (Thermo Fisher Scientific, Rockford IL, USA) and a Gel Doc Imaging system equipped with a XRS camera and Quantity One analysis software (Bio-Rad). GAPDH (Trevigen Biotechnology Gaithersburg, Maryland, USA) was used as a protein loading control (Baldea et al., 2013).

### 2.5. Statistical Analysis

The statistical significance of the difference between treated and control groups was evaluated with two-way ANOVA, followed by Dunnett's multiple test using GraphPad Prism version 4.00 for Windows (GraphPad Software, San Diego, California, USA, http://www.graphpad.com). A *p* value < 0.05 was considered statistically significant.

## 3. Results

### 3.1. The Antimicrobial Activity

One of the goals of our study was to determine the antimicrobial activity of the *Equisetum arvense* L. extract obtained from nonpolluted areas of Bihor County. The results regarding the antimicrobial activity of *Equisetum arvense* L. extract evaluated by disc diffusion test are summarized in Table 1. *Equisetum arvense* L. extract exhibited an antibacterial effect on gram-positive bacteria except the clinical isolate of *Streptococcus agalactiae* GBBHS (group B beta-hemolytic streptococcus) and *Staphylococcus epidermidis*, the last one being a methicilin-resistant strain. Our extract was the most effective against *Streptococcus pneumoniae* and against the clinical isolate of *Streptococcus pyogenes*. No antimicrobial activity was found on *Candida albicans* or on gram-negative bacteria. In MIC determinations, the lowest concentration of *Equisetum arvense* L. extract, at which there was visible growth, was 25 mg/ml for *Staphylococcus aureus* (ATCC 29213) and human isolate *Staphylococcus aureus* and 12.5 mg/ml for streptococcal strains: *Streptococcus pneumoniae* (ATCC 49619), *Streptococcus pyogenes group A*, and *group G beta-hemolytic Streptococcus.* Subcultures on Columbia agar + 5% sheep blood (BioMérieux) from tubes with 25 mg/ml *Equisetum arvense* L. extract for staphylococci and 12.5 mg/ml for streptococcal were positive. These values represent the MICs. Subcultures from tubes with 50 mg/ml for staphylococci and 25 mg/ml for streptococcal were negative; these values represent the minimum bactericidal concentrations (MBCs).

### 3.2. The Antioxidant, Anti-Inflammatory, and Antiapoptotic Activities on HUVECs

Surface marker analysis performed by flow cytometry showed the presence of ICAM-1, CD105, CD29, and CD73 thus suggesting that the cells used in experiment exhibit endothelial functional parameters.

**Figure 1:**
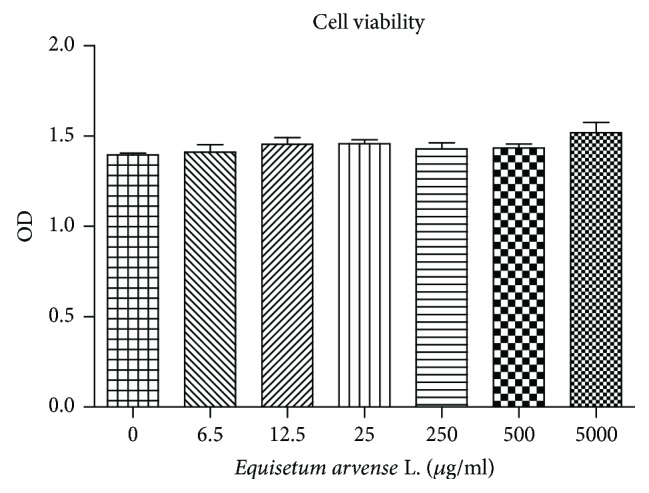
Cell viability testing. *Equisetum arvense* L. extract was tested across a range of concentrations up to 5000 *μ*g/ml in HUVECs. The viability graphs did not show significant changes in cell viability even at high doses (5000 *μ*g/ml) (mean values ± standard deviation, *n* = 3).

The toxicity of the *Equisetum arvense* L. extract tested on HUVECs did not lead to any significant changes in cell viability even when high doses (5000 *μ*g/ml) were used (Figure 1).

**Figure 2:**
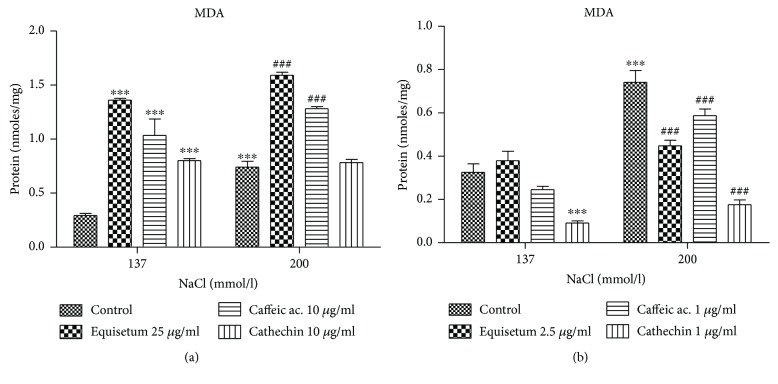
Malondialdehyde levels evaluated in endothelial cells exposed to hyperosmotic stress and pretreated with two doses of *Equisetum arvense* L. extract. (a) The MDA levels after exposure to hyperosmotic stress and pretreatment for 24 h with high doses of *Equisetum arvense* L. extract, caffeic acid, and cathechin. (b) MDA levels after exposure to hypertonic conditions and low doses of *Equisetum arvense* L. extract, caffeic acid, and cathechin. The statistical significance of the difference between the treated and control groups was evaluated with two-way ANOVA, followed by Dunnett's multiple test; ^###^*p* < 0.001, treated versus untreated cells in hypertonic medium; ^∗∗∗^*p* < 0.001, hypertonic medium versus the parameters in normotonic conditions.

The hypertonic solution has a prooxidant effect on the lipids of endothelial cells, as shown by the increased lipid peroxide levels (*p* < 0.0001) in the cells exposed to high concentrations of saline solutions (Figure 2(a)). At a dose of 25 *μ*g/ml, the *Equisetum arvense* L. extract has a prooxidant effect, increasing the lipid peroxide levels, both in the basic conditions (normotonic solution) and in the hypertonic solutions (*p* < 0.001). The caffeic acid and cathechin significantly decreased the lipid peroxide levels compared to the extract (*p* < 0.001) (Figure 2(a)). When using lower doses of the same substances, we found that, in normotonic conditions, the lipid peroxide level was not significantly affected by the treatment with *Equisetum arvense* L. while in hypertonic conditions, the extract had an antioxidant effect, significantly decreasing the lipid peroxidation (*p* < 0.001). The best protection of endothelial cells against hyperosmotic stress was exerted by the low concentration of cathechin (*p* < 0.001) (Figure 2(b)).

**Figure 3:**
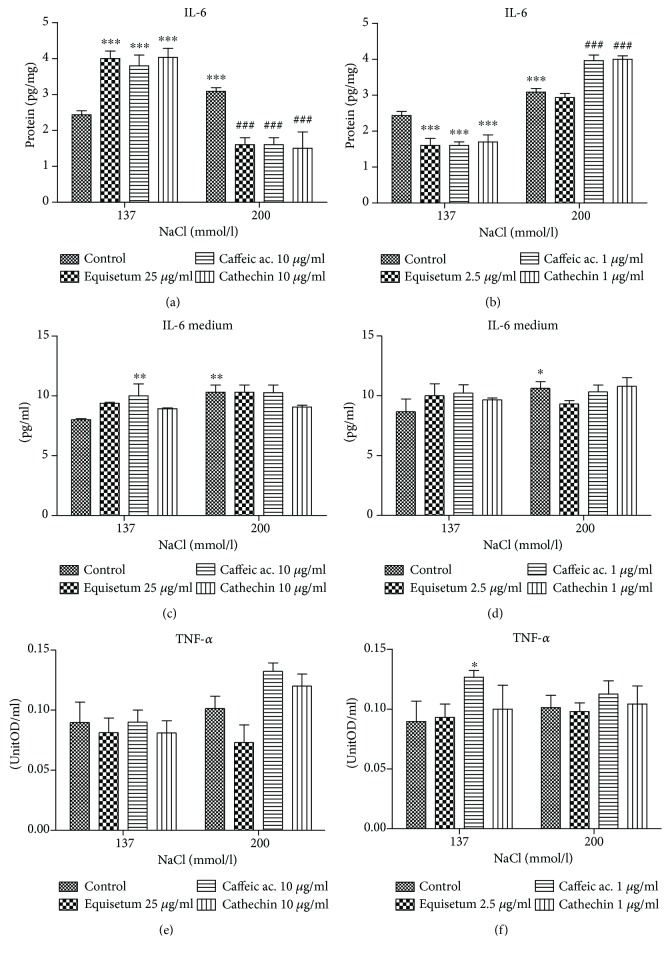
Proinflammatory cytokines evaluated in endothelial cells exposed to hyperosmotic stress and pretreated with two doses of *Equisetum arvense* L. extract. (a) The IL-6 secretion in cell lysates after exposure to hyperosmotic stress and pretreatment for 24 h with high doses of *Equisetum arvense* L. extract, caffeic acid, and cathechin. (b) IL-6 level in cell lysates after exposure to hypertonic conditions and low doses of the three substances tested. (c, d) IL-6 concentrations in medium after exposure to normotonic and hypertonic conditions and treatment with the three substances tested. (e) TNF-*α* level in cell lysates in hypertonic conditions and pretreatment with high doses of *Equisetum arvense* L. extract, caffeic acid, and cathechin. (f) TNF-*α* secretion in cell lysates in hypertonic conditions and pretreatment with low doses of *Equisetum arvense* L. extract, caffeic acid, and cathechin. The statistical significance of the difference between treated and control groups was evaluated with two-way ANOVA, followed by Dunnett's Multiple test; ^###^*p* < 0.001, treated versus untreated cells in hypertonic medium; ^∗^*p* < 0.05, ^∗∗^*p* < 0.01, and ^∗∗∗^*p* < 0.001, hypertonic medium versus the parameters in normotonic conditions.

In order to evaluate the extract effect on inflammation, the levels of IL-6, in both intracellular and the growth medium, and TNF-*α* were quantified (Figures 3(a)–3(d)). For assessing the role of the NF-*κ*B pathway activation in inflammation induced by hypertonic conditions and the impact of low doses of natural compounds, Western blot was performed. After treatments, the protein levels of the total NF-*κ*B protein and the active form, pNF-*κ*B, and also I*κ*B and pI*κ*B were estimated.

Intracellular levels of IL-6, evaluated in endothelial cell lysates, significantly increased in hypertonic conditions (*p* < 0.01) (Figures 3(a) and 3(b)). The preadministration of *Equisetum arvense* L. or caffeic acid and cathechin, in high doses, amplified the increasing of IL-6 secretion in normotonic conditions, in parallel with the generation of oxidative stress (*p* < 0.001). In hypertonic medium, the pattern is different; the three tested substances had a stronger anti-inflammatory effect (*p* < 0.001). The lower doses of the three studied compounds had no proinflammatory effects in normal conditions, but caffeic acid and cathechin were proinflammatory under hypertonic conditions. The low dose of *Equisetum arvense* L. extract had no significant effect on the IL-6 levels in hypertonic medium (*p* > 0.05). In hypertonic conditions, we registered an increased concentration of IL-6 in the medium compared to the normotonic situation (*p* < 0.01). The administration of the high dose of *Equisetum arvense* L. and caffeic acid had no significant effect on the IL-6 levels found in the growth medium (*p* > 0.05) (Figure 3(c)).

On the contrary, the pretreatment with 10 *μ*g/ml of cathechin reduced significantly IL-6 secretion in the medium. The lower doses of *Equisetum arvense* L., caffeic acid, and cathechin had no effect on IL-6 secretion found in the growth media (Figure 3(d)).

The hypertonic conditions had no significant effect on the TNF-*α* secretion compared to normotonic medium (Figures 3(e) and 3(f)). The *Equisetum arvense* L. extract did not influence the TNF-*α* levels, in both normotonic and hypertonic conditions. The same effect was exerted by cathechin in both environmental situations. Caffeic acid, in high dose, increased the TNF-*α* protein level in endothelial cell lysates (*p* < 0.01). The lower doses of the three compounds did not influence significantly the TNF-*α* secretion in both experimental conditions (Figure 3(f)).

**Figure 4:**
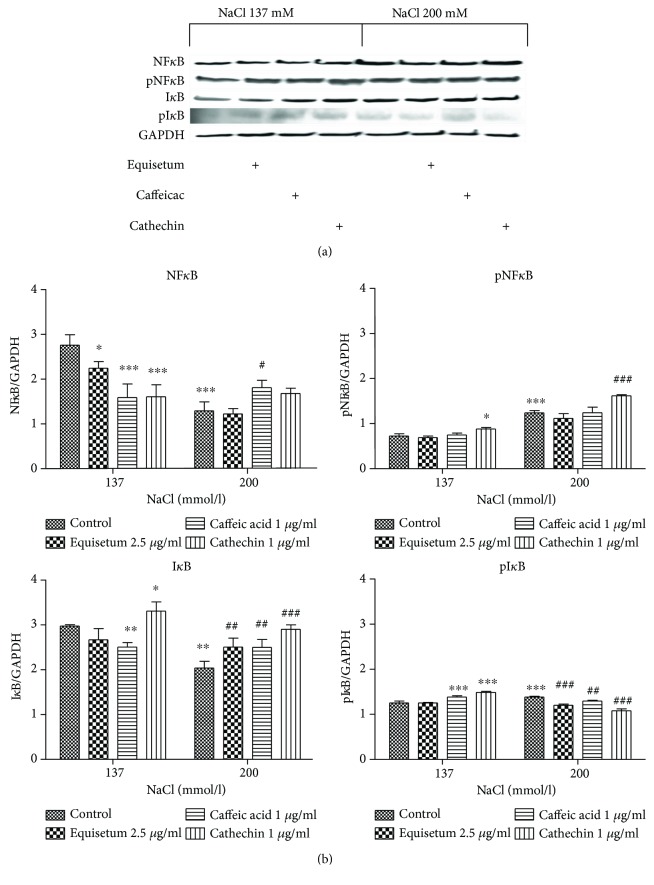
The expressions of NF-*κ*B, pNF-*κ*B, I*κ*B, and pI*κ*B in endothelial cells exposed to hyperosmotic stress and pretreated with low dose of *Equisetum arvense* L., caffeic acid, and cathechin. (a) Comparative Western blot images showing expressions of NF-*κ*B, pNF-*κ*B, I*κ*B, and pI*κ*B in HUVECs exposed to normotonic and hypertonic conditions and treated with the three compounds. (b) Image analysis of Western blot bands was done by densitometry; results were normalised to GAPDH. Western blot images: 1 = cells in normotonic medium, 2 = cells in normotonic medium treated with *Equisetum arvense* L., 3 = cells in normotonic medium treated with caffeic acid, 4 = cells in normotonic medium treated with cathechin, 5 = cells in hypertonic medium, 6 = cells in hypertonic medium treated with *Equisetum arvense* L., 7 = cells in hypertonic medium treated with caffeic acid, and 8 = cells in hypertonic medium treated with cathechin. Graphical representation of the quantitative Western blot results for HUVECs in the two conditions. Two-way ANOVA, followed by Dunnett's multiple test, was used to evaluate the statistical significance of differences in the mean values of the measured parameters. Each bar represents mean ± standard deviation (*n* = 3), ^#^*p* < 0.05, ^##^*p* < 0.01, and ^###^*p* < 0.001, treated versus untreated cells in hypertonic medium; ^∗^*p* < 0.05, ^∗∗^*p* < 0.01, and ^∗∗∗^*p* < 0.001, hypertonic medium versus the parameters in normotonic conditions.

The effects of normotonic/hypertonic medium and natural extract administration on expressions of NF-*κ*B, pNF-*κ*B I*κ*B, and pI*κ*B proteins were evaluated by Western blot analysis (Figure 4(a)). Phosphorylation of p65/Rel A at Ser 536 regulates activation, nuclear localization, protein-protein interaction, and transcriptional activity. The key factor in the NF-*κ*B pathway activation involves Ikappa kinase (I*κ*K) complex, and I*κ*B is an inhibitor which sequesters NF*κ*B dimers in the cytoplasm in an inactive state. In normotonic medium, the treatment with low dose of *Equisetum arvense* L. extract (*p* < 0.05) and low doses of caffeic acid (*p* < 0.001) and cathechin (*p* < 0.001) diminished the expression of NF-*κ*B compared to controls, in parallel with increasing of I*κ*B expression, especially after cathechin administration (*p* < 0.05) (Figure 4(b)).

In hypertonic conditions, NF-*κ*B level decreased compared to normotonic medium (*p* < 0.001) and increased only after caffeic acid treatment (*p* < 0.01). In hypertonic medium, all natural compounds used in the experiment increased the expression of I*κ*B, mostly cathechin in low dose (*p* < 0.001). The best inhibitory effect on I*κ*B activation was noticed in cells exposed to cathechin (*p* < 0.001), then after *Equisetum arvense* L. (*p* < 0.01) and caffeic acid (*p* < 0.01) treatments.

The active form of NF-*κ*B represents a modest part of NF-*κ*B protein, both in normotonic and in hypertonic medium, as illustrated in Figure 4(b). Hypertonicity induced a significant increase of pNF-*κ*B protein expression (*p* < 0.001), effect amplified by cathechin administration (*p* < 0.001) which exerted an inhibitory effect on pI*κ*B (*p* < 0.001). *Equisetum arvense* L. and caffeic acid had also a strong inhibitor effect on activation of I*κ*B in hypertonic medium while in normotonic conditions, the same compounds enhanced its expression (*p* < 0.001 and *p* < 0.01, resp.).

**Figure 5:**
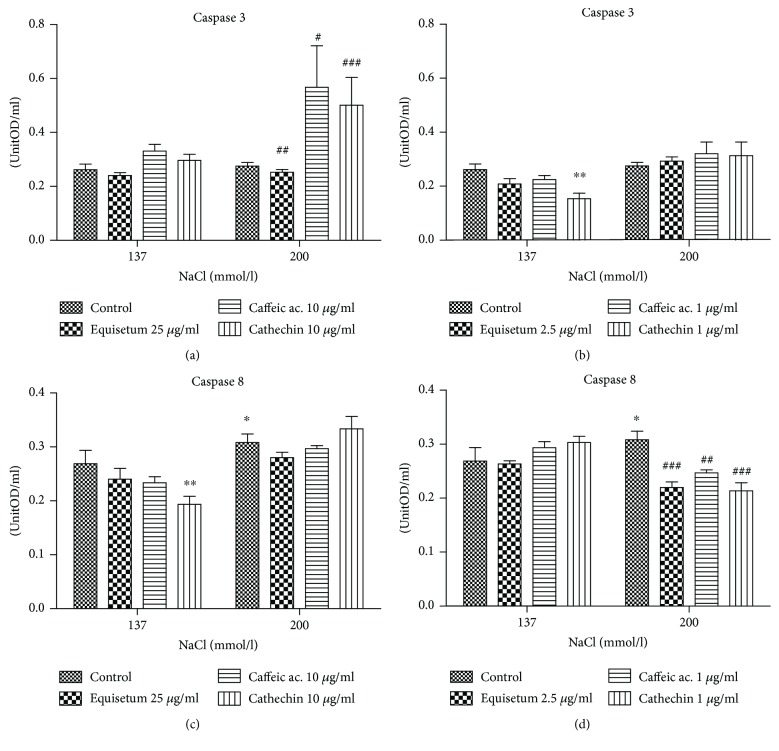
The activities of caspase-3 and caspase-8 evaluated in endothelial cells exposed to hyperosmotic stress and pretreated with two doses of *Equisetum arvense* L. extract. (a) The caspase-3 activity in endothelial cells after exposure to hyperosmotic stress and pretreatment for 24 h with high doses of *Equisetum arvense* L. extract, caffeic acid, and cathechin. (b) The caspase-3 activity in endothelial cells after exposure to hypertonic conditions and low doses of *Equisetum arvense* L. extract, caffeic acid, and cathechin. (c) The caspase-8 activity in endothelial cells after exposure to hyperosmotic stress and pretreatment for 24 h with high doses of *Equisetum arvense* L. extract, caffeic acid, and cathechin. (d) The caspase-8 activity in endothelial cells after exposure to hypertonic conditions and low doses of *Equisetum arvense* L. extract, caffeic acid, and cathechin. The statistical significance of the difference between treated and control groups was evaluated with two-way ANOVA, followed by Dunnett's multiple test, ^#^*p* < 0.05, ^##^*p* < 0.01, and ^###^*p* < 0.001, treated versus untreated cells in hypertonic medium; ^∗^*p* < 0.05 and ^∗∗^*p* < 0.01, hypertonic medium versus the parameters in normotonic conditions.

In our experimental design, the hypertonic medium did not induce apoptosis compared to normotonic conditions (Figure 5). In the normotonic system, preincubation with low dose of cathechin decreased significantly the caspase-3 activity (*p* < 0.01) while in hypertonic medium, the same compound did not influence the enzyme activity (Figure 5(b)). Caffeic acid and cathechin in high doses were proapoptotic and enhanced the activation of enzyme (*p* < 0.01) in hypertonic conditions while the normotonic medium did not change the enzyme activity (Figure 5(a)).

Preadministration of 10 *μ*g/ml of cathechin had an antiapoptotic effect decreasing caspase-8 activity in endothelial cells exposed to normotonic conditions (*p* < 0.01) (Figure 5(c)). Higher doses of *Equisetum arvense* L., caffeic acid, and cathechin did not influence the caspase-8 activity in hypertonic conditions (*p* > 0.05) but, in lower doses, decreased the caspase-8 activity, especially *Equisetum arvense* L. and cathechin, and thus the induction of apoptosis in the same conditions (*p* < 0.001) (Figures 5(c) and 5(d)).

## 4. Discussion

In previous studies, it has been demonstrated that plants, fruits, and vegetables, due to their phytochemicals composition, have antioxidant, antimicrobial, and anti-inflammatory actions and are used in medicine and nutrition including the cosmetic industry [31–33]. In our study, the *Equisetum arvense* L. extract exhibited an antibacterial effect on *Streptococcus pneumoniae* and the clinical isolate of *Streptococcus pyogenes.*

Studies over the past decade attested the *in vitro* antimicrobial activity *Equisetum arvense* L. extract, but with some conflicting results. Milovanović et al. [34] described an antifungal effect on *Candida albicans* while in our study, we did not obtain any inhibitor effect on *Candida albicans* strain. Our results are in agreement with previous findings reported by Uslu et al. [35] and Čanadanović-Brunet et al. [36]. De Oliveira et al. also demonstrated that *Equisetum arvense* L. extract at a concentration of 50 mg/mL was effective against *Staphylococcus aureus*, *Staphylococcus epidermidis*, *Streptococcus mutans*, *Candida albicans*, *Candida tropicalis*, and *Candida glabrata*, microorganisms involved in oral infections [37]. Protective effects of *Equisetum arvense* L. extract on *Staphylococcus aureus* and gram-negative bacteria are also described, both in the aqueous and alcoholic extract, with better response in the case of the alcoholic extract [38, 39]. Radulovic et al. [40] stated that a 1 : 10 dilution of the essential oil obtained by hydrodistillation of aerial parts of *Equisetum arvense* L. was enough for its strong antimicrobial activity against *Staphylococcus aureus*, *Escherichia coli*, *Klebsiella pneumoniae*, *Pseudomonas aeruginosa*, *Salmonella enteritidis*, and fungi: *Aspergillus niger* or *Candida albicans*. The effects were explained by a composition rich in the essential oil of the plant extract which inactivated the microbial adhesion proteins and transport proteins and induced the rupture of the microbial membrane [41]. In addition, the phenolic compounds reduced the ROS generation induced by bacterial lipopolysaccharides or *Candida albicans* due to trapping free radicals directly or scavenging them through reactions with antioxidant enzymes [42].

However, other studies showed no effect of the aqueous extract on *Staphylococcus aureus* and *Escherichia coli* [36, 43]. One of the explanations for these conflicting results could be the extraction parameters used for obtaining the plant extract which influence the chemical content and the biological activities, including the antimicrobial activity, [35] or different content in phenols [44]. From a quantitative point of view, the flavonoids and polyphenols levels were higher in the first months of the plant's growth (May, June) compared to the harvest period (August, September). During the month of May, the quantity of flavonoids increased up to 0.72% compared to September, when the values decreased to 0.13% [28]. In similar studies, plants coming from other regions have been shown to have a much lower antioxidant activity [45].

In our extract, the total content of polyphenols and flavonoids was high, as it resulted from findings performed with spectrophotometric techniques [28]. Using HPLC techniques, the large quantities of vanillic, caffeic, ferulic, syringic, synaptic, and gallic acids as phenolic acids and quercetin, epicathechin, cathechin, rutin, naringenin, and myricetin as flavonoids [46] were found. These parameters were in agreement with other findings [47]. The results showed also an *in vitro* strong antioxidant activity of the extracts obtained from the sterile stems of *Equisetum arvense* L. Using the FRAP technique, the value determined was 84.160 *μ*mol Trolox equivalent/g DW (TE), using CUPRAC technique, 49.2 *μ*mol Trolox equivalent/g DW (TE), and using DPPH method, 87.3% [46].

Osmotic stress is involved as a pathogenetic mechanism in many cardiovascular diseases and is associated with the efflux of water from the cells and shrinking of cells. In this process, the activity of water channels (aquaporins) and electrolyte transporters, and also the accumulation of osmolytes within the cells [48] for protection of proteins and subcellular structures, are involved.

It is known that under severe hypertonic conditions or when the adaptation mechanisms are outdated, the cells enter to apoptosis and die [49]. Moreover, it seems that apoptosis is associated with persistence of cell shrinkage due to inhibition of protein transport in normal cells [50] or deprivation of glutamine, which functions as compatible osmolyte. In our study, the hypertonic conditions increased only oxidative stress and inflammation and did not influence the apoptosis. Practically, the hypertonicity did not increase the caspase activity compared to normotonic medium; one explanation is probably related to the adaptation process to osmotic stress. The dynamic adaptation to hypertonic environment involved a biphasic response, one early, in seconds or minutes, and another late, in hours and days. The reduction of cellular volume is rapidly corrected by the activation of ion transport systems including Na^+^-K^+^-2Cl^−^ cotransporter, Na^+^/H^+^ exchanger, and the Cl^−^/HCO_3_^−^ exchanger which lead to intracellular accumulation of ions and increasing of cell volume [51]. In the later phase, the synthesis of compatible osmolytes including neutral amino acids, polyols such sorbitol, and also methylamine such as betaine increased and they substituted the inorganic ions and protected the cell from apoptosis [52]. In this process, the major role is played by TonEBP/NFAT5, a transcription factor involved in the transactivation of “osmoprotective genes” including taurine transporter, aldose reductase, betaine/GABA transporter, and *myo*-inositol transporter [17]. In addition, activating protein- (AP-) 1 induced by JNK and p38 signaling pathways acts as a central switch to convert extracellular signals into genetic responses [53].

Cytoskeletal remodeling, antioxidant response, and unfolded protein response are other important mechanisms of osmoadaptation. In addition, hyperosmotic stress was found to directly induce TNF-*α*, IL-1*β*, IL-6, and IL-8 secretion from different cells [19]. In these mechanisms, the activation of protein kinase R (PKR) under hyperosmotic stress and stimulation of NF-*κ*B p65 phosphorylation at serine 536 and consequently the induction of inflammatory NF-*κ*B p65-responsive genes were involved [54]. Moreover, it was demonstrated that protein kinase R directly phosphorylated I*κ*B*α*. This is an inhibitor which can mask the nuclear localization of NF*κ*B and sequester NF*κ*B dimers in an inactive state in cytoplasm. I*κ*B phosphorylated is then ubiquitinated and degraded by the proteasome, freeing NF-*κ*B to enter the nucleus and induce expression of inflammatory genes [55]. A higher amount of pNF-*κ*B was obtained when cells were exposed to hypertonic medium alone or in association with cathechin administration, suggesting that cathechin addition increased both the total NF-*κ*B protein and its activation. The active form of I*κ*B protein diminished in cells exposed to *Equisetum arvense* L., caffeic acid, and cathechin in parallel with increasing of IL-6 levels and NF-*κ*B activation, especially after cathechin administration.

In the experiment, the caspase activities were evaluated only at 24 h after incubation in the hypertonic environment, when the whole adaptation process took place. However, at this time, both oxidative stress and inflammation induced by hypertonicity were maintained at an increasing rate compared to normotonic conditions. In general, oxidative stress is caused by the intracellular accumulation of reactive oxygen species (ROS) or a disturbance of the cellular redox state and can cause intracellular proteins, lipids, and DNA damage. ROS have also been implicated as major mediators of stress-induced signaling as a response to various kinds of environmental stress including osmotic stress. In hyperosmotic stress, the activation of membrane-associated phospholipase A_2_ and generation of prostaglandins including arachidonic acid is one of the mechanisms involved in the production of redox misbalancing. Phospholipase A_2_ activates the NADPH oxidase and increases the superoxide anion formation [56]. In addition, xanthine oxidase, nitric oxide (NO) synthase, and heme oxygenases are other sources of intracellular ROS. Anion superoxide in turn induces the activation of protein tyrosine kinases implicated in the regulation of volume-sensitive osmolyte channels and thus helps to maintain cellular homeostasis [57].

In our study, the low doses of the three compounds reduced the lipid peroxidation and protected the cells against stress induced by hypertonic conditions and diminished the activity of caspase-8 and consequently the apoptosis triggered by extrinsic mechanism. In the same conditions, caffeic acid and cathechin, in low concentrations, increased IL-6 secretion and NF-*κ*B activation and diminished pI*κ*B in cells and did not influence the caspase-3 activity. The high doses of the three substances had the opposite effect on inflammation and oxidative stress. Thus, *Equisetum arvense* L., cathechin, and caffeic acid exerted prooxidant effect and reduced the IL-6 secretion in cells. In addition, caffeic acid and cathechin in high concentrations induced the secretion of TNF-*α* in the endothelial cells in parallel with prooxidant and proapoptotic effects suggesting the cellular toxicity of high doses of polyphenols.

The different effect on IL-6 secretion can be explained by double functions of IL-6 in cardiovascular systems. IL-6 is secreted in different cells of cardiovascular structures including macrophages, fibroblasts, and endothelial cells as a response to the exposure to cytokines (IL-1, TNF-*α*), oxidative stress, and angiotensin II and after vascular injury [58]. IL-6 can activate the target cell through a classical signaling pathway after binding to IL-6 receptor (IL-6R) on the cell surface and also by “trans-signaling” mechanism due to soluble IL-6-IL-6R*α* complex. This complex initiates the IL-6 signaling [59] and activates the signal transducer and the activator of transcription (STAT) involved in the transcription of target genes. Classic signaling is involved in the activation of anti-inflammatory pathways on target cells while the trans-signaling pathway leads to activation of the immune system by the recruitment of monocytes in the inflamed area and transition from acute to chronic inflammation. It seems that IL-6 suppress the level of proinflammatory cytokines in acute inflammatory response without affecting the secretion of anti-inflammatory cytokines [60].

Our results confirmed the findings of Bessa Pereira et al. [61] regarding the antibacterial effect and Četojević-Simin et al. [7] concerning the antioxidant properties of *Equisetum arvense* L. extract. The anti-inflammatory activity of the extract demonstrated by Do Monte et al. [62] in wounds and arthritis confirmed the multiple valences of this natural compound.

## 5. Conclusions

*Equisetum arvense* L. extract exhibited antibacterial effects on pathogenic gram-positive cocci but had no effect on gram-negative bacteria and *Candida albicans.* The three compounds tested exert the antioxidant activity and diminished the activity of caspase-8 only in low concentrations, protecting the cells exposed to hyperosmotic stress. In high doses, *Equisetum arvense* L., cathechin, and caffeic acid had prooxidant effects, induced apoptosis, and decreased IL-6 secretion.

The modulatory effects of *Equisetum arvense* L. extract on endothelial cells exposed to hypertonic medium are different and depend on the concentration used. These experimental findings suggest that *Equisetum arvense* L. administered in low doses may be a new therapeutic approach to decrease the enhanced oxidative stress and apoptosis associated with hypertonic conditions. In addition, *Equisetum arvense* L. extract exhibits antimicrobial activity against gram-positive cocci. However, further more complex studies on different cell lines with multiple doses or exposure times are necessary to prove its clinical potency.

## Figures and Tables

**Table 1:** The antimicrobial activity of *Equisetum arvense* L. extract.

		Zone of growth inhibition (in mm diameter)									
Extract	Conc	*Staphylococcus aureus* ATCC 25923	*Streptococcus pneumoniae* ATCC 49619	*E. coli* ATCC 25922	*Pseudomonas aeruginosa* ATCC 27853	*Candida albicans* ATCC 90029	*Streptococcus pyogenes*	*Streptococcus agalactiae*	*Group G beta-hemolytic streptococcus*	*Staphylococcus epidermidis*	*Staphylococcus aureus*
*Equisetum arvense* L.	100 mg/ml	8	11	6	6	6	9	6	8	6	8
Penicillin	10 U	30	26	Not tested	Not tested	Not tested	32	33	34	20	22
Vancomycin	30 *μ*g	18	23	Not tested	Not tested	Not tested	22	20	23	Not tested	Not tested
Ofloxacin	5 *μ*g	27	18	30	19	Not tested	19	20	18	16	24
Meropenem	10 *μ*g	30	31	29	28	Not tested	Not tested	Not tested	Not tested	Not tested	Not tested
Fluconazole	25 *μ*g	Not tested	Not tested	Not tested	Not tested	32	Not tested	Not tested	Not tested	Not tested	Not tested
Distilled water		6	6	6	6	6	6	6	6	6	6
